# miR-9-5p Inhibits Skeletal Muscle Satellite Cell Proliferation and Differentiation by Targeting IGF2BP3 through the IGF2-PI3K/Akt Signaling Pathway

**DOI:** 10.3390/ijms21051655

**Published:** 2020-02-28

**Authors:** Huadong Yin, Haorong He, Xiaoxu Shen, Jing Zhao, Xinao Cao, Shunshun Han, Can Cui, Yuqi Chen, Yuanhang Wei, Lu Xia, Yan Wang, Diyan Li, Qing Zhu

**Affiliations:** Farm Animal Genetic Resources Exploration and Innovation Key Laboratory of Sichuan Province, Sichuan Agricultural University, Chengdu 611130, China; yinhuadong@sicau.edu.cn (H.Y.); hehaorong@stu.sicau.edu.cn (H.H.); shenxiaoxu@stu.sicau.edu.cn (X.S.); zhaojing@stu.sicau.edu.cn (J.Z.); caoxinao@stu.sicau.edu.cn (X.C.); hanshunshun@stu.sicau.edu.cn (S.H.); cuican123@stu.sicau.edu.cn (C.C.); chenyuqi@stu.sicau.edu.cn (Y.C.); weiyuanhang@stu.sicau.edu.cn (Y.W.); xlaza@sicau.edu.cn (L.X.); wangyan519@sicau.edu.cn (Y.W.); diyanli@sicau.edu.cn (D.L.)

**Keywords:** miR-9-5p, IGF2BP3, IGF2-PI3K/Akt, skeletal muscle satellite cell, proliferation, differentiation

## Abstract

MicroRNAs are evolutionarily conserved, small non-coding RNAs that play critical post-transcriptional regulatory roles in skeletal muscle development. We previously found that miR-9-5p is abundantly expressed in chicken skeletal muscle. Here, we demonstrate a new role for miR-9-5p as a myogenic microRNA that regulates skeletal muscle development. The overexpression of miR-9-5p significantly inhibited the proliferation and differentiation of skeletal muscle satellite cells (SMSCs), whereas miR-9-5p inhibition had the opposite effect. We show that insulin-like growth factor 2 mRNA-binding protein 3 (IGF2BP3) is a target gene of miR-9-5p, using dual-luciferase assays, RT-qPCR, and Western Blotting, and that it promotes proliferation and differentiation of SMSCs. In addition, we found that IGF2BP3 regulates IGF-2 expression, using overexpression and knockdown studies. We show that Akt is activated by IGF2BP3 and is essential for IGF2BP3-induced cell development. Together, our results indicate that miR-9-5p could regulate the proliferation and differentiation of myoblasts by targeting IGF2BP3 through IGF-2 and that this activity results in the activation of the PI3K/Akt signaling pathway in skeletal muscle cells.

## 1. Introduction

Skeletal muscle is the most abundant tissue in our body, accounting for approximately 40%–60% of body mass in adult humans and animals [1]. The loss of function of skeletal muscle, especially through distorted muscle differentiation, causes various diseases, including cancer and diabetes [2]. Additionally, skeletal muscle is the most economically valuable tissue in meat-producing animals, and its quantity and quality determine the efficiency of production [3]. Skeletal muscle is composed of post-mitotic multinucleated myofibroblasts [4]. Its development is a tightly regulated, multi-step process in which mesodermal precursor myoblasts differentiate into myoblasts and then fuse to form multinucleated myofibers [5]. Myogenesis is orchestrated through various myogenic transcription factors (including DNA, peptides and noncoding RNAs) and various signaling pathways [6].

In keeping with the emerging interest in the molecular mechanisms that control skeletal muscle development, a new and exciting aspect of gene regulation has gained attention in recent years, with the discovery of microRNAs (miRNAs) [7,8]. miRNAs are a class of small noncoding RNAs, of approximately 22 nucleotides, encoded by endogenous genes. They function by degrading or repressing the translation of target mRNAs, by binding to the 3′ untranslated region (UTR) of target mRNAs, regulating gene expression levels [9]. It is well known that miRNAs are involved in diverse roles in fundamental biological processes, including cell proliferation, differentiation, apoptosis, and tumorigenesis [10]. 

Initially, miR-9 was shown to be a critical regulator of organ development, and function in neurogenesis [11]. Subsequently, however, miR-9 has been reported to be highly expressed in many types of malignant tumors [12]. Recently, research in many tumor types found that miR-9 can be silenced by CpG island hypermethylation, indicating that miR-9 possesses tumor suppressor features [13]. In a previous study, we found that the miR-9-5p is abundantly expressed in the breast muscle of embryonic and 7-week-old chickens [14]. These findings suggested that miR-9-5p may be involve in myogenesis. However, the molecular function and target gene of miR-9-5p, through which it exercises its control over the proliferation and differentiation of skeletal muscle cells, remains unclear.

Through analysis of our previous miRNA-Seq and RNA-Seq data (NCBI accession number PRJNA516545), we identified insulin-like growth factor 2 mRNA-binding protein 3 (IGF2BP3) as the target gene of miR-9-5p. IGF2BP3 is a member of the insulin-like growth factor mRNA binding protein (IGFBP) family, which can bind with insulin-like growth factor 2 (IGF2) to promote cell development [15]. Interestingly, IGF2BP3 has been reported to play a role in chicken skeletal muscle development by regulating IGF2 [16]. The aim of the present study was to test our hypothesis that miR-9-5p participated in skeletal muscle myogenesis via the downregulation of IGF2BP3 in chickens.

## 2. Results

### 2.1. Expression of miR-9-5p in Skeletal Muscle Satellite Cells (SMSCs) 

**Figure 1:**
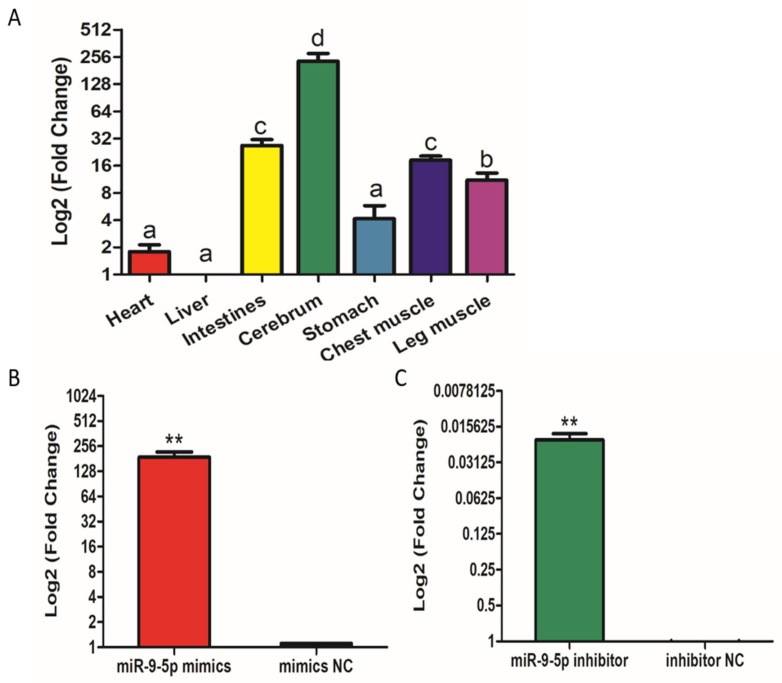
Expression of miR-9-5p in skeletal muscle satellite cells (SMSCs). (**A**) The expression of miR-9-5p in tissues of broiler chickens, the mRNA level in liver as a control. (**B**,**C**) The expression of miR-9-5p in SMSCs was monitored using qRT-PCR following transfection with a miR-9-5p inhibitor or mimic. The mRNA levels were log-transformed, and data are presented as mean ± SEM (standard error of the mean) (*n* = 6*)*. * *p* < 0.05; ** *p* < 0.01 vs. NC (negative control).

Firstly, we identified the expression levels of miR-9-5p in different tissues. The results show that miR-9-5p is relatively highly expressed in leg and chest muscle tissues, suggesting that miR-9-5p may be related to muscle development (Figure 1A). To determine the role of miR-9-5p in the proliferation and differentiation of chicken SMSCs, we constructed a miR-9-5p inhibitor, and a mimic, to suppress expression and simulate over-expression. When SMSCs were transfected with the miR-9-5p mimic, the expression of miR-9-5p increased by over 180 times (Figure 1B), whereas a 50-fold decrease in expression was found in SMSCs treated with the inhibitor (Figure 1C). These results confirm that they could be used in further experiments.

### 2.2. MiR-9-5p Inhibits the Proliferation of SMSCs

**Figure 2:**
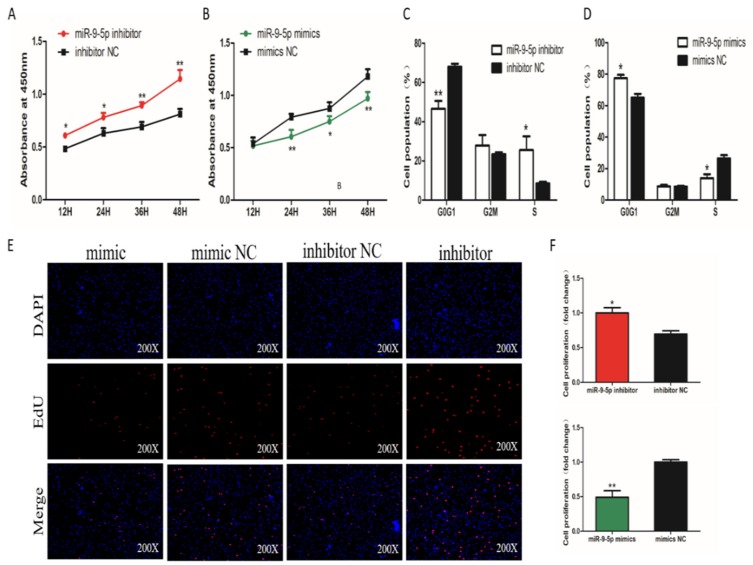
Effect of miR-9-5p on the proliferation of SMSCs. (**A**,**B**) Cell growth curves, as measured using the CCK-8 assay following transfection with a miR-9-5p mimic, inhibitor or negative control (NC) in SMSCs. (**C**,**D**) Cell cycle analysis of SMSCs 48 h after the overexpression or inhibition of miR-9-5p. (**E**) The results of the EdU assay for SMSCs transfected with a miR-9-5p mimic, inhibitor, or negative control, where EdU (red) fluorescence is used as an indicator of proliferation, and nuclei are indicated by DAPI (blue) fluorescence. Photomicrographs were taken using a 200× magnification. (**F**) The proliferation rates of SMSCs with miR-9-5p overexpression and inhibition. The data are expressed as mean ± SEM (*n* = 6). * *p* < 0.05; ** *p* < 0.01 vs. NC.

To assess the potential roles of miR-9-5p in SMSC proliferation, the Cell Counting Kit 8 (CCK-8) assay was used to detect the proliferation activity. The results show that SMSCs transfected with the miR-9-5p inhibitor had significantly higher proliferation than control cells (Figure 2A). In contrast, the proliferation activity was significantly inhibited in cells overexpressing miR-9-5p (Figure 2B). Cell cycle analysis, using flow cytometry, revealed that miR-9-5p inhibition elevated the number of cells in S phase and reduced the percentage of cells in G1/G0 phase (Figure 2C). The number of cells in the S phase was decreased and the population of G0/G1 cells was increased in SMSCs transfected with miR-9-5p mimics (Figure 2D). We also examined SMSCs proliferation using the EdU assay and found that the number of EdU-positive cells in the proliferation period was significantly increased by miR-9-5p knockdown and decreased by miR-9-5p overexpression (Figure 2E,F). Together, these results strongly suggest that miR-9-5p can inhibit the proliferation of chicken SMSCs.

### 2.3. MiR-9-5p Inhibits the Differentiation of SMSCs

**Figure 3:**
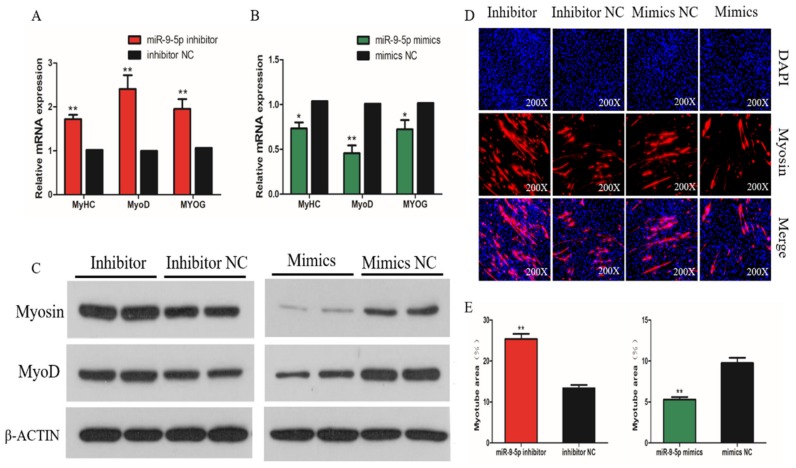
Effect of miR-9-5p on differentiation of SMSCs. (**A**,**B**) The relative expression of myogenic determination (MyoD), myosin heavy chain (MyHC), and myogenin (MyoG) mRNA in SMSCs allowed us to differentiate for 48h with miR-9-5p inhibition or overexpression. (**C**) The protein levels of myogenic marker genes after the inhibition and overexpression of miR-9-5p, from Western Blot. (**D**) Representative images of immunofluorescent staining of differentiated SMSCs (200x magnification). Myosin: red, a molecular marker of myogenesis; DAPI: blue, cell nuclei; Merge: the fusion of SMSCs into primary myotubes. (**E**) The myotube area (%) after the transfection of miR-9-5p mimics, miR-9-5p inhibitor, or NC. The data are expressed as mean ± SEM (*n* = 6). * *p* < 0.05; ** *p* < 0.01 vs. *NC.*

To determine the effect of miR-9-5p in SMSC differentiation, we measured the mRNA expression of three myogenic differentiation marker genes: myogenic determination 1 (MyoD1), myogenin (MyoG), and myosin heavy chain (MyHC). The results show that the overexpression of miR-9-5p significantly reduced the mRNA expression of MyoD1, MyoG, and MyHC, whereas their levels were significantly raised in miR-9-5p knockdown SMSCs (*p* < 0.05; Figure 3A,B). The same effects on MyoG and MyHC protein abundance were found in SMSCs when treated with the miR-9-5p mimic or inhibitor (Figure 3C). The immunofluorescence images of myosin suggest that the knockdown of miR-9-5p promoted SMSC differentiation, whereas the overexpression of miR-9-5p appears to inhibit myotube formation during SMSC differentiation (Figure 3D). In addition, after the inhibition of miR-9-5p, the myotube area increased significantly, while the opposite was true with miR-9-5p inhibition (Figure 3E). Overall, these results suggest that miR-9-5p inhibits the differentiation of chicken SMSCs. 

### 2.4. MiR-9-5p Targets the IGF2BP3 Gene Directly

**Figure 4:**
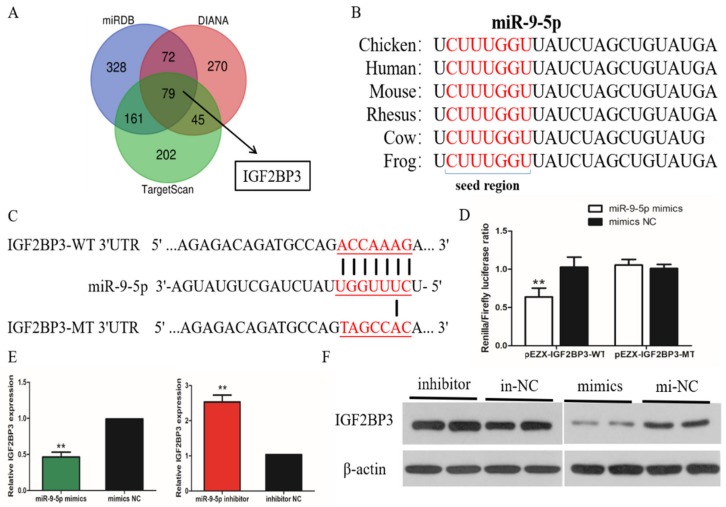
MiR-9-5p targets directly IGF2BP3 gene. (**A**) The prediction of target genes using TargetScan, miRDB and Diana. (**B**) The seed region of miR-9-5p. (**C**) The dual-luciferase reporter gene (pEZX-FR02) with wild type (pEZX-IGF2BP3-WT) or mutant (pEZX-IGF2BP3-MT). (**D**) The luciferase assays were performed by the co-transfection of wild-type or mutant IGF2BP3 3’ UTR with a miR-9-5p mimic or mimic-NC in SMSCs. (**E**,**F**) After transfection of miR-9-5p mimics, miR-9-5p inhibitor, or NC, the expression of IGF2BP3 was determined by q-PCR and Western Blot. The data are expressed as mean ± SEM (*n* = 6). * *p* < 0.05; ** *p* < 0.01.

To explore the regulatory mechanism by which miR-9-5p inhibits chicken SMSC proliferation and differentiation, we analyzed the MiR-9-5p target gene levels in relation to the proliferation and differentiation status of skeletal muscle cells. Three online software tools, TargetScan, miRDB and Diana, were used to predict target genes of miR-9-5p. The combined results gave 79 genes as the most likely target genes, including IGF2BP3 (Figure 4A). Subsequent analysis found that the 3’ UTR of IGF2BP3 is a potential binding site for miR-9-5p. This site is conserved among human, chimp, mouse, rat, rabbit, and pig species (Figure 4B). To verify the possible target relationship between miR-9-5p and IGF2BP3 in chicken SMSCs, we constructed a dual-luciferase reporter gene (pEZX-FR02) with a wild type (pEZX-IGF2BP3-WT) or mutant (pEZX-IGF2BP3-MT) IGF2BP3 3’UTR sequence at the 3’ end of the firefly luciferase (Figure 4C). Firefly luciferase activity was decreased in DF-1 cells co-transfected with the dual-luciferase reporter gene and miR-9-5p mimic compared with control (*p* < 0.05), while no changes were observed in cells co-transfected with the mutated reporter (*p* > 0.05; Figure 4D). We determined the mRNA expression and protein abundance of IGF2BP3 in SMSCs treated with miR-9-5p mimic or inhibitor. The results show that IGF2BP3 mRNA abundance is downregulated by the overexpression of miR-9-5p, and upregulated when miR-9-5p is inhibited (Figure 4E,F). These results all indicate that there is a direct target relationship between miR-9-5p and IGF2BP3.

### 2.5. IGF2BP3 Promotes the Proliferation of SMSCs

**Figure 5:**
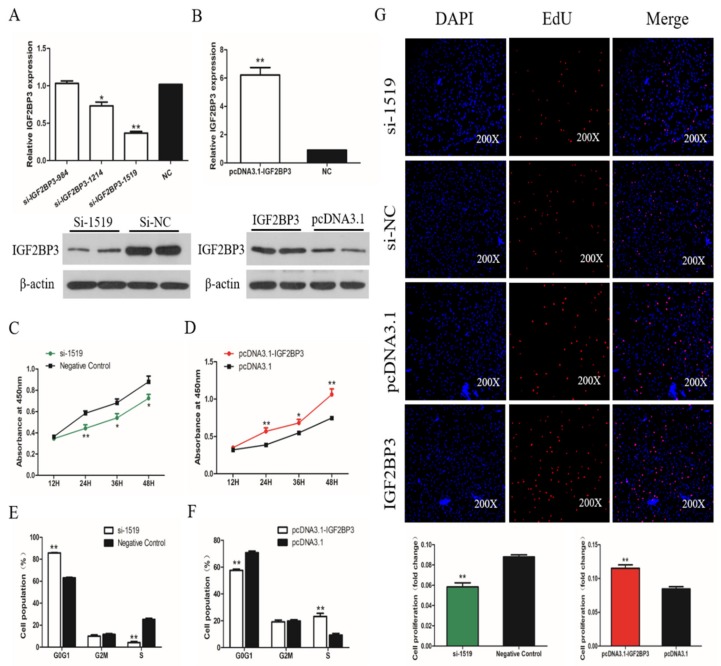
The effect of IGF2BP3 on the proliferation of SMSCs. (**A**,**B**) Screening IGF2BP3 interfering fragments and verifying overexpression efficiency by qPCR and Western Blot. The data are presented as mean ± SEM (*n.* = 6*)*. The Student’s *t*-test was used to compare expression levels among different groups. * *p* < 0.05; ** *p* < 0.01 vs. NC. (**C**,**D**) Cell growth curves, as measured from CCK-8 assays, following transfection with si-IGF2BP3, pcDNA3.1-IGF2BP3, or negative control in SMSCs. (**E**,**F**) Cell cycle analysis of SMSCs 48 h after the overexpression and inhibition of IGF2BP3. (**G**) The results of the EdU assay for SMSCs transfected with si-IGF2BP3, pcDNA3.1-IGF2BP3, or negative control, where EdU (red) fluorescence is used as an indicator of proliferation and nuclei are indicated by DAPI (blue) fluorescence. Micrographs were taken using 200× magnification, and the proliferation rates of SMSCs cells were measured with IGF2BP3 overexpression and inhibition. The data are expressed as mean ± SEM (*n.* = 6). * *p* < 0.05; ** *p* < 0.01 vs. NC.

To determine the role of IGF2BP3 in the proliferation and differentiation of SMSCs, we constructed and validated an overexpression vector and siRNA targeting IGF2BP3 (Figure 5A,B). We used the CCK-8 assay to verify the following results: The cellular proliferation viability was increased significantly in SMSCs transfected with the IGF2BP3 overexpression vector, and significantly reduced in cells expressing IGF2BP3 siRNA (Figure 5C,D). On the over-expression of IGF2BP3 in SMSCs, we found that the proportion of cells in the G1/0 phase was decreased and the proportion of cells in S phase was increased. The opposite trend occurred in SMSCs treated with siRNA targeting IGF2BP3 (Figure 5E,F). In addition, the trend of the number of EDU-positive cells after IGF2BP3 increased or decreased also accorded with this conclusion (Figure 5G). These results suggest that IGF2BP3 promotes the proliferation of chicken SMSCs.

### 2.6. IGF2BP3 Promotes the Differentiation of SMSCs

**Figure 6:**
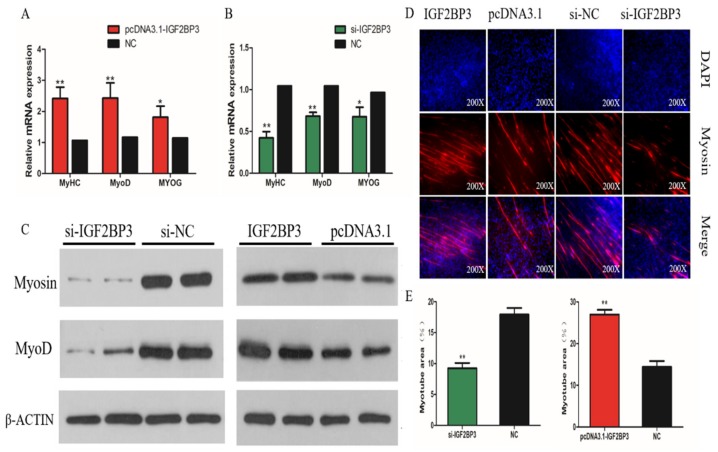
Effect of IGF2BP3 on differentiation of SMSCs. (**A**,**B**) The relative expressions of MyoD, MyHC, and MyoG mRNA in SMSCs allowed to differentiate for 48h. (**C**) The Western Blot results for protein levels of myogenic marker genes after inhibition and overexpression of IGF2BP3. (**D**) The Representative images of immunofluorescent staining of differentiated SMSCs (200x). Myosin: red, a molecular marker of myogenesis; DAPI: blue, cell nuclei; Merge: the fusion of SMSCs into primary myotubes. (**E**) The Myotube area (%) after the transfection of si-IGF2BP3, pcDNA3.1-IGF2BP3 or negative control. The data are expressed as mean ± SEM (*n* = 6). * *p* < 0.05; ** *p* < 0.01 vs. NC.

To elucidate the role of IGF2BP3 in the differentiation of chicken SMSCs, we over-expressed or knocked down IGF2BP3 levels, respectively. We found that MyoD1, MyoG, and MyHC mRNA expression increased, following the overexpression of IGF2BP3, while the opposite was true following the inhibition of IGF2BP3 (Figure 6A,B). The Western Blot results show that MyoG and MyHC protein abundance was upregulated after the overexpression of IGF2BP3, and downregulated following IGF2BP3 silencing (Figure 6C). Immunofluorescence assays further confirm these results, demonstrating that the number of Myosin-positive cells increased following the overexpression of IGF2BP3, and decreased following the inhibition of IGF2BP3 (Figure 6D). The changes in the myotube area, observed after the interference and overexpression of IGF2BP3 are also consistent with this trend (Figure 6E).

### 2.7. IGF2BP3 is Involved in the IGF2-PI3k/AKt Pathway. 

**Figure 7:**
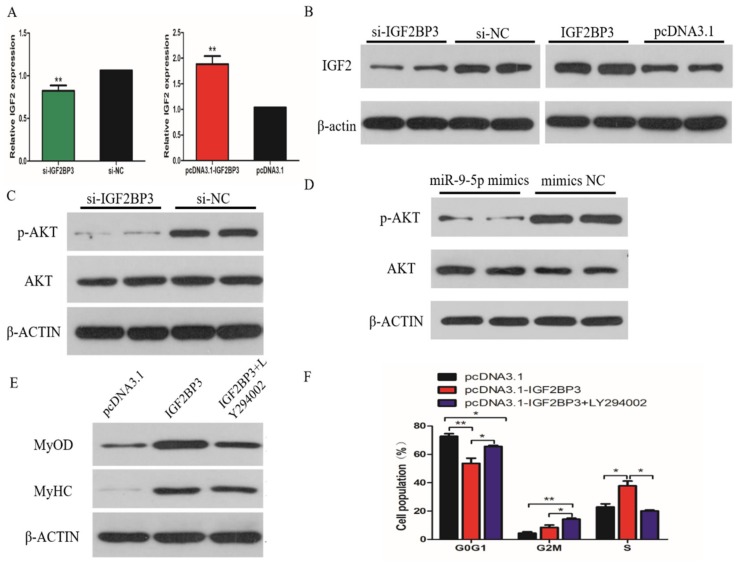
IGF2BP3 is involved in the IGF2-PI3K/Akt pathway. (**A**,**B**) The expression of IGF2 in SMSCs, following transfection of si-IGF2BP3, pcDNA3.1-IGF2BP3 or NC, determined by q-PCR and Western Blot. (**C**,**D**) The Western Blot results showing the levels of phosphor-Akt (p-Akt) and Akt in IGF2BP3-inhibited and miR-9-5p-overexpressed cells. (**E,F**) The effect of pcDNA3.1-IGF2BP3 on the proliferation and differentiation of SMSC cells in the absence or presence of LY294002. The data are expressed as mean ± SEM (*n* = 6). * *p* < 0.05; ** *p* < 0.01 vs. NC.

The previous study reported that IGF2BP3 is involved with the phosphatidylinositol3-kinase (PI3K) pathway through modulating IGF-2 in glioblastoma. We therefore hypothesized that miR-9-5p may regulate the proliferation and differentiation of SMSCs through the IGF2-PI3K/Akt signal pathway. To verify this, we determined the mRNA and protein level of IGF-2 in SMSCs when IGF2BP3 was over-expressed or inhibited. The results show that increased IGF2BP3 expression correlates with increased IGF-2 transcript and protein levels, and we observed a significant reduction in IGF-2 mRNA and protein abundance upon siRNA-mediated IGF2BP3 knockdown (Figure 7A,B). Similarly, we found that the levels of phosphor-Akt were reduced upon treatment with IGF2BP3 siRNA in SMSCs without a corresponding change in the levels of total Akt (Figure 7C). Meanwhile, the same results were obtained after promoting the expression of miR-9-5p in SMSCs (Figure 7D). To further validate the importance of this pathway in IGF2BP3-induced SMSCs proliferation and differentiation, we assayed the growth and development of IGF2BP3-overexpressing cells under conditions where the PI3K/AKT signal pathway was pharmacologically inhibited. The results show that exogenous IGF2BP3 increased cell proliferation and myotube formation, but that the proliferation and differentiation of these IGF2BP3-overexpressing cells was significantly reduced when treated with LY294002 (the PI3K inhibitor; Figure 7E,F). 

## 3. Discussion

The normal growth and maintenance of skeletal muscle is essential for animal and human health as it plays crucial roles in structural support, maintaining posture, the control of motor movements, energy storage, and whole-body metabolism [17,18]. Chicken is considered to be one of the best models to study skeletal muscle formation in vertebrates because the developmental anatomy of chicken skeletal muscles is very similar to that of mammals [19]. Additionally, identifying the transcription factors that regulate skeletal muscle growth and development could also be important in improving the yield and quality of chicken meat. 

Skeletal muscle development is a well-coordinated biological process that is regulated by evolutionarily conserved networks of myogenic transcription factors [20]. Recent studies have shown that miRNA is involved in the regulation of mammalian skeletal muscle development. In this study, the results of CCK-8 assays show that the downregulation or upregulation of miR-9-5p repressed or promoted proliferation of SMSCs, respectively. This suggests that miR-9-5p can inhibit the proliferation of chicken SMSCs. We strengthened these observations by examining the cell cycle in SMSCs similarly subject to miR-9-5p modulations, using flow cytometry. Additionally, we found that miR-9-5p also inhibited the differentiation of SMSCs, by analyzing critical positive regulators of myogenesis (MyoD1, MyoG and MyHC) and myotube formation. Our results are similar to those reported by Spillane et al. [21], which showed that miR-9 overexpression decreases cell viability and increases apoptosis in MCF-7 breast cancer cells.

The identification of target genes is critical in the functional characterization of miRNAs. It is well known that miRNAs bind to sites located in the 3′ untranslated regions (3′ UTRs) of mRNAs to downregulate their expression [22]. The functions and molecular targets of miR-9-5p have been identified in several cancer cells. For instance, miR-9-5p suppresses prostate cancer progress by targeting StarD13 [23], and miR-9 downregulates CDX2 expression in gastric cancer cells [24]. In this study, we identified putative target genes of miR-9-5p using three types of analysis software (TargetScan, miRDB and Diana) and found that the 3′ UTR of IGF2BP3 matched the mature sequence of miR-9-5p, and this binding site in the miR-9-5p of chicken is the same as that found in humans. In addition, a previous report showed that miR-9-5p was decreased following an acute exercise bout of healthy men, and which increased in different muscle wasting diseases [25]. Next, we used the dual luciferase assay to confirm IGF2BP3 as a target of miR-9-5p, showing the overexpression or inhibition of miR-9-5p resulted in lesser or greater expression of IGF2BP3, respectively. To our knowledge, our findings reveal, for the first time, both a myogenic regulation role for miR-9-5p and a novel target (IGF2BP3).

The IGF2BP family, which has three members (IGF2BP1, IGF2BP2, and IGF2BP3) are known to bind to IGF-2 mRNA and thereby regulate its stability and translation [26,27]. IGF2BP3 was first discovered in this role because of its high expression in pancreatic carcinoma [28]. Subsequent studies have also found a high expression of IGF2BP3 in various tumor types [29,30,31]. Lin et al. [16] found that IGF2BP3 has a significantly higher expression in the skeletal muscle of seven-week-old normal chicken than in dwarf chicken, indicating a potential role in chicken skeletal muscle growth and development. In this study, the CCK-8 assay, EdU assay, and flow cytometry were used to determine the effect of IGF2BP3 on SMSCs proliferation, and our results demonstrate that IGF2BP3 can significantly promote proliferation. Meanwhile, the overexpression of IGF2BP3 can significantly upregulate the expression of myogenic factors that were related to myoblast differentiation, and accelerate myotube formation, which indicates that IGF2BP3 can suppress myoblast differentiation.

The PI3K/Akt pathway is a critical cellular pathway involved in many cell functions, including cell survival, cell differentiation, cell growth, and cell motility [32]. Skeletal muscle myogenesis and muscle hypertrophy have also been found to be strictly regulated by PI3K/Akt signaling [33]. Previous studies have reported that the dysregulated expression of miRNAs affects the PI3K/Akt signaling pathway to alter skeletal muscle growth and development. For example, miR-146b-3p suppresses chicken myoblast proliferation and differentiation and promotes apoptosis by directly suppressing both the PI3K/Akt pathway and the Myod family inhibitor domain containing protein [34], miRNA-432 is a potent inhibitor of myogenesis which targets E2F3 and P55PIK in muscle cells [35], and miRNA-21 is involved in skeletal muscle development and regulates PI3K/Akt/mTOR signaling by targeting the TGFβI gene [36]. In this study, we showed that IGF2BP3 expression is positively correlated with IGF-2 levels using knockdown and overexpression studies. The mitogenic effects of the IGF-2 can be mediated through the PI3K pathway. We found that IGF2BP3 can activate the PI3K pathway because the signaling cascade in this pathway was negatively affected by the siRNA-mediated knockdown of IGF2BP3. Finally, we established that IGF2BP3 was involved in the PI3K/Akt pathway using a pharmacological inhibitor of this pathway, which reduced IGF2BP3-induced proliferation in SMSCs. Our results are consistent with the work of Suvasini et al. [27], which concludes that IMP3 can activate the PI3K/Akt pathway through the translational activation of IGF-2 in glioblastomas. 

**Figure 8:**
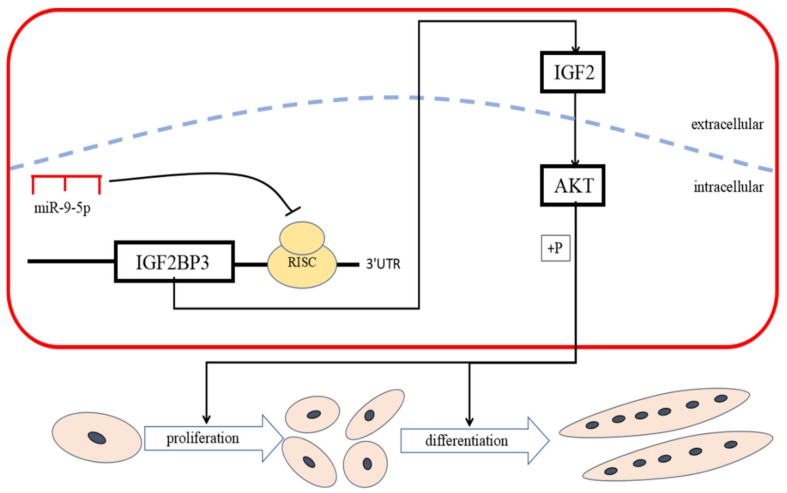
miR-9-5p inhibits skeletal muscle cell proliferation and differentiation by targeting IGF2BP3 through the IGF2-PI3K/Akt signaling pathway.

In conclusion, we reveal a role for miR-9-5p as a novel miRNA that plays an active role in the proliferation and differentiation of chicken SMSCs by inhibiting IGF2BP3 through the IGF-2/PI3K/Akt pathway (Figure 8). To our knowledge, our study reports, for the first time, that miR-9-5p functions as a negative regulator of myogenic differentiation. In addition, a better understanding of the molecular regulatory mechanisms of miR-9-5p SMSCs development will provide a theoretical basis for the RNA treatment of muscle diseases.

## 4. Materials and Methods

### 4.1. Ethics Standards

All animal experimental procedures in this study were approved by the Animal Welfare Committee of Sichuan Agriculture University (6 May 2019), and the assurance number is 2019-0AT07.

### 4.2. Animals

The specialized broiler chickens (ROSS 308) were purchased from the Xinjin Yunda Poultry Breeding Cooperative (Chengdu, China)

### 4.3. Cells Cultures

The detailed procedures of collection and culture of chicken skeletal muscle satellite cells (SMSCs) were followed by the previously report by Han et al. [37]. The chicken blast fibroblast cell line DF-1 was cultured in Dulbecco’s Modified Eagle Medium (DMEM; Sigma, St. Louis, MO, USA), 10% fetal bovine serum (FBS, Gibco, Grand Island, NY, USA), and 0.5% penicillin-streptomycin (Solarbio, Beijing, China) at 37°C and 5% CO2 with saturating humidity, and the medium was refreshed every 24 h.

### 4.4. RNA Oligonucleotides and Plasmids Construction

The MiR-9-5p inhibitor, inhibitor NC, miR-9-5p mimic, mimic NC, and IGF2BP3 siRNA in this study were designed and synthesized by a commercial company (RiboBio, Guangzhou, Guangdong, China), and the detailed sequences are shown in Table 1.

### 4.5. Cell Transfection 

Interference or overexpression experiments were performed when SMCSs density reached 70% to 80%. All transient transfections were performed with Lipofectamine 3000 reagent (Invitrogen, Carlsbad, CA, USA) according to the manufacturer’s protocol with at least three replications. Then, the cells were collected after transfection for RNA and protein extraction.

### 4.6. Extraction of RNA and Real-Time PCR (RT-PCR)

Total RNA was extracted using TRIzol reagent (Invitrogen) according to manufacturer’s instructions. The integrity and concentration of RNA in samples was measured using the Thermo Scientific™ NanoDrop Lite (Thermo, Waltham, MA, USA). Total RNA was stored at −80 °C. The reverse transcription of mRNA was performed using PrimeScript RT Master Mix Perfect Real Time (Takara, Dalian, China), and the reverse transcription reactions for miRNA were performed using the One Step miRNA cDNA Synthesis Kit as per the manufacturer’s instructions (HaiGene, Haerbin, Heilongjiang, China).

Real-time PCR primers were designed using Primer Premier 6 and are listed in Table 2. An abundance of mRNA for each gene was measured using CFX96-TouchTM real-time PCR detection system (Bio-Rad, Hercules, CA, USA). The reaction volume for real-time PCR was 10 μL and consisted of 1 μL cDNA, 0.5 μL reverse and forward primers (per gene), 3 μL double-distilled water, and 5 μL TB Green™ Premix Ex Taq™ II (Takara). All reactions were performed in triplicate. The relative gene expression was determined by the 2 ^–ΔΔCT^ method.

### 4.7. CCK-8 Assay

Cell proliferation was measured using the Cell Count Kit-8 (Meilunbio, Shanghai, China) according to the manufacturer’s instructions with 6 repetitions per run. Absorbance was measured at 450 nm using Thermo Scientific™ Varioskan LUX (Thermo, San Jose, CA, USA). The experiments were repeated five times and performed 0, 12, 24, 48, and 72 h following transfection, respectively.

### 4.8. EdU Assay

Following transfection, cells were incubated with EdU medium according to the manufacturer’s instructions and were then washed using PBS (phosphate buffer saline). Next, the cells were stained using a C10310 EdU Apollo In Vitro Imaging Kit (RiboBio, Guangzhou, China) after fixing. Three regions were randomly selected using a fluorescence microscope to assess the number of stained cells.

### 4.9. Immunofluorescence

According to manufacturer’s instructions, the cells were fixed using 4% paraformaldehyde (RiboBio) on glass coverslips and washed three times using PBS for 3 min. Fixed cells were permeabilized using 0.5% Triton X-100 for 20 min at room temperature and blocked with goat serum for 30 min. Next, cell diluted primary antibody was added and incubated overnight at 4°C. The slides were diluted using PBST (0.05% Tween 20 + PBS) three times and diluted secondary antibody was added, and incubated at 37°C for 1 h. Next, the cell nuclei were stained using DAPI (4’, 6-diamidino-2-phenylindole; RiboBio) in the dark for 5 min. Images were taken using a fluorescence microscope (Olympus, Melville, NY, USA).

### 4.10. Western Blot

Detailed experimental methods for Western Blot analysis are described in-depth by Han et al. (Han et al., 2019a). Antibodies used for experiments included: anti-Myosin (Santa Cruz Biotechnology, CA, USA; 1:200 dilution), anti-MyoD (Santa Cruz Biotechnology; 1:500 dilution), anti-caspase-3 (Santa Cruz Biotechnology; 1:1000 dilution), anti-caspase-9 (Santa Cruz Biotechnology; 1:1,000 dilution), anti-AKT, anti-p-AKT (Santa Cruz Biotechnology; 1: 1000 dilution), anti-IGF2BP3, anti-IGF2 (abclonal, Wuhan, China; 1:1000 dilution), and β-actin (ZENBIO, Beijing, China, 1: 5000 dilution). β-actin was used as a loading control.

### 4.11. Luciferase Reporter Assay

Fragments of miR-9-5p, including the binding site of IGF2BP3, were amplified and inserted into pEZX-FR02 vectors (GeneCopoeia, Amaranth Drive Germantown, Maryland, USA) at the 3’ end of the Firefly Luciferase gene using restriction enzymes BsiWI and XhoI (TaKaRa) and T4 DNA ligase (pEZX- IGF2BP3-WT). Mutant pEZX- IGF2BP3-MT was generated by mutating complementary to the seed region of miR-9-5p using mutagenic primers. All constructs were verified by sequence analysis.

### 4.12. Prediction of Target Genes

The target genes of miR-9-5p were predicted using TargetScan (http://www.targetscan.org/vert_71/), miRDB (http://mirdb.org/), and Diana (http://www.microrna.gr/microT-CDS).

### 4.13. Statistical Analysis

Statistical analyses were performed using SPSS 19.0 Statistical software (SPSS, Inc., Chicago, IL, USA). Each experiment was repeated three times. A one-way analysis of variance or paired t-test was used to test statistical significance between groups. The data are presented as least square means ± standard error of the mean (SEM). The student’s *t*-test was used for two group comparisons and ANOVA, and Tukey’s test was used for multiple group comparisons. Differences were considered significant at the *p* < 0.05 level. 

## Figures and Tables

**Table 1:** RNA oligonucleotides in this article.

Name	Sequence (5′-3′)
miR-9-5p mimic	UCUUUGGUUAUCUAGCUGUAUGA
Negative mimic	UUGUACUACACAAAAGUACUG
miR-9-5p inhibitor	UCAUACAGCUAGAUAACCAAAGA
Negative inhibitor	CAGUACUUUUGUGUAGUACAA
Si-IGF2BP3-984	GCUGCUGAGAAACCAAUUATT UAAUUGGUUUCUCAGCAGCTT
Si-IGF2BP3-1214	GCAGGACUUGACACUCUAUTT AUAGAGUGUCAAGUCCUGCTT
Si-IGF2BP3-1519	CCUUGGCAGUUGGAGCUAUTT AUAGCUCCAACUGCCAAGGTT
Si-NC	UUCUUCGAACGUGUCACGUTT ACGUGACACGUUCGGAGAATT

For IGF2BP3 overexpression plasmid construction, the coding sequence fragments of IGF2BP3 was cloned into pcDNA3.1 (+) vector using T4 DNA ligase (TakaRa; Tokyo, Japan) following the restrictive endonuclease digestion of HindIII and KpnI restriction enzyme (TakaRa). Then, the successfully constructed vectors were termed as pcDNA3.1- IGF2BP3 after confirming by sequencing.

**Table 2:** Primers used for quantitative real-time PCR.

Genes	Forward primer (5′-3′)	Reverse primer (5′-3′)
β-actin	GTCCACCGCAAATGCTTCTAA	TGCGCATTTATGGGTTTTGTT
MyoG	TACAGCGACCAACAGTACGC	TCTGCATTGTTTCCATCCTG
MyoD1	AGCACTACAGTGGCGACTCA	GGCCGCTGTAATCCATCA
MYHC	GAAGGAGACCTCAACGAGATGG	ATTCAGGTGTCCCAAGTCATCC
IGF2BP3	GCTGCTGCTGCTTCATATCCAC	CCTGCTTGCCAATAATAGCTCCA
IGF2	AGGATCAACCGTGGCATTGT	TCTGACTTGACGGACTTGGC
miR-9-5p	TCTTTGGTTATCTAGCTGTATGA	CAGGTCCAGTTTTTTTTTTTTTT
U6	GGGCCATGCTAATCTTCTCTGTA	CAGGTCCAGTTTTTTTTTTTTTT
